# Salt Stress Promotes Abscisic Acid Accumulation to Affect Cell Proliferation and Expansion of Primary Roots in Rice

**DOI:** 10.3390/ijms221910892

**Published:** 2021-10-08

**Authors:** Yingying Huang, Jiahao Zhou, Yuxiang Li, Ruidang Quan, Juan Wang, Rongfeng Huang, Hua Qin

**Affiliations:** 1 Biotechnology Research Institute, Chinese Academy of Agricultural Sciences, Beijing 100081, China; huangyingying_cool@163.com (Y.H.); 18210521037@163.com (J.Z.); liyx0929@163.com (Y.L.); quanruidang@caas.cn (R.Q.); wangjuan@caas.cn (J.W.); rfhuang@caas.cn (R.H.); 2 National Key Facility of Crop Gene Resources and Genetic Improvement, Beijing 100081, China

**Keywords:** primary root, salt, ABA, cell proliferation, cell expansion, rice

## Abstract

The primary root is the basic component of the root system and plays a key role in early seedling growth in rice. Its growth is easily affected by environmental cues, such as salt stress. Abscisic acid (ABA) plays an essential role in root development, but the molecular mechanism underlying ABA-regulated root growth in response to salt stress remains poorly understood. In this study, we report that salt stress inhibits primary root elongation and promotes primary root swelling. Moreover, salt stress induces the expression of ABA-responsive genes and ABA accumulation in the primary root, revealing that ABA plays an essential role in salt-modulated root growth. Transgenic lines of *OsSAPK10-OE* and *OsABIL2-OE*, which constitutively express *OsSAPK10* or *OsABIL2*, with enhanced or attenuated ABA signaling, show increased and decreased sensitivity to salt, correspondingly. Microscopic analysis indicates that salt and ABA inhibits cell proliferation and promotes cell expansion in the root apical meristem. Transcriptome analysis showed that ABA induces the expression of *EXPANSIN* genes. Further investigations indicate that ABA exerts these effects largely through ABA signaling. Thus, our findings deepen our understanding of the role of ABA in controlling primary root growth in response to salt stress, and this knowledge can be used by breeders to cultivate rice varieties suitable for saline–alkali land.

## 1. Introduction

As a belowground organ, roots anchor plants and take up water and nutrients from the soil. Root development therefore strongly affects plant growth and productivity [1]. Rice is a staple crop for half of the world’s population and improving the root system of rice is conducive to increasing the yield of crops and their ability to cope with adverse environments [1,2,3]. Salinity stress is one of the major constraints on plant growth, affecting rice productivity worldwide, and roots are the primary target site for perception of salt stress signals which could act as an early warning system for the plant [4,5]. The rice root system consists of a primary root, lateral roots, and adventitious roots [6]. The primary root initiates during embryogenesis and develops shortly after germination, and plays a key role in seedling establishment [7]. The growth of the primary root is maintained by cell proliferation in the root apical meristem (RAM) and cell elongation in the elongation zone, plant hormones playing an important role in this process [8,9,10].

Abscisic acid (ABA) is known as a plant stress hormone and plays an important role in root growth [11,12,13]. Studies in *Arabidopsis* have uncovered a core ABA signaling pathway. ABA is perceived by pyrabactin resistance 1 (PYR1)/PYR1-like (PYL)/regulatory components of ABA receptor (RCAR), which in turn interacts with the subclass A type 2C protein phosphatases (PP2Cs), leading to the activation of SNF1-related protein kinase 2 (SnRK2s). As a result, SnRKs phosphorylate and activate basic leucine zipper (bZIP) transcription factors to regulate the expression of ABA-responsive genes [14,15,16,17]. Studies in *Arabidopsis* have shown that ABA has biphasic effects on primary root elongation, depending on its concentration. Typically, low concentrations of ABA stimulate primary root elongation, whereas high concentrations inhibit it [18,19]. However, the underlying mechanism in this process is largely unknown.

In addition to regulating primary root elongation, ABA also functions in root swelling. Exogenous application of 10 μM ABA leads to root apical swelling, mainly due to the expansion of root apical cortical cells [20]. EXPANSINs are a class of cell wall-loosening proteins that are involved in cell wall extension and cell growth and underly many developmental processes in plants [21,22,23]. EXPANSINs play important roles in root growth and development, and several EXPANSINs have been investigated in rice. Mutations in the root hair-specific *EXPANSIN* genes *OsEXPA17* and *OsEXPA30* display the inhibition of root hair elongation [24]. OsEXPA8 positively regulates root system architecture by facilitating cell extension [21,25]. *OsEXPA10* is an Al-inducible *EXPANSIN* gene and is required for root cell elongation [26]. *OsEXPB2* is a root-predominant gene with a key role in root-hair formation and its expression is suppressed by ABA [27]. Multipass (OsMPS) is a direct upstream regulator of *OsEXPA4*, *OsEXPA8*, *OsEXPB2*, *OsEXPB3*, and *OsEXPB6*, and its expression is induced by ABA and cytokinin [28]. Based on these observations, we hypothesized that EXPANSINs might be involved in ABA-modulated root elongation and swelling.

In this study, we report that salt stress promotes the accumulation of ABA in primary roots, thereby inhibiting cell proliferation and promoting cortical cell expansion in the root meristem, resulting in short and swollen primary roots. Transcriptome analysis indicates that ABA regulates numerous processes including cell wall organization or biogenesis. Interestingly, we found that *EXPANSIN* genes are facilitated by ABA. Our study helps uncover the potential molecular mechanism of salt-modulated primary root elongation and swelling, providing insight into the coordination of environmental and developmental signals during root development.

## 2. Results

### 2.1. Salt Stress Promotes ABA Accumulation to Inhibit Root Elongation and Promote Root Swelling

**Figure 1:**
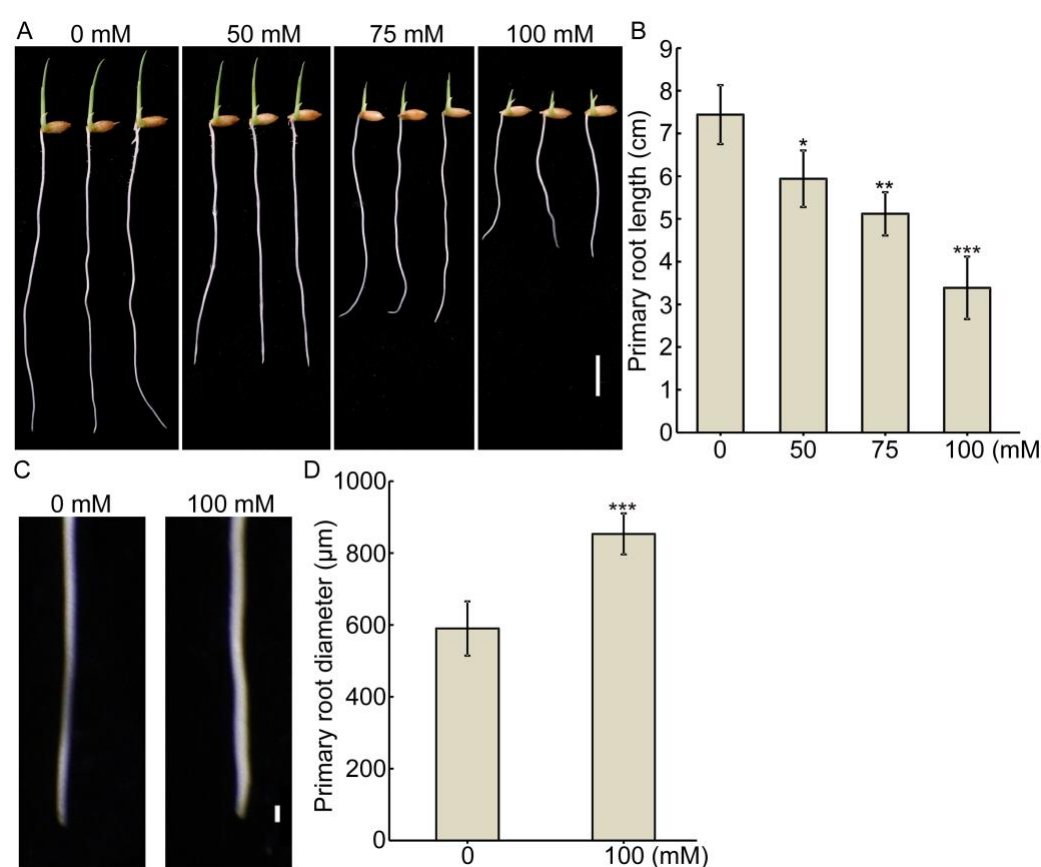
Salt stress inhibits root elongation and promotes root swelling. (**A**,**C**) Root phenotypes of (Nipponbare) Nip seedlings with various concentrations of NaCl treatment. Rice seedlings were grown under normal conditions for 4 days in the various concentrations of NaCl aqueous solution. Bar = 1 cm (**A**) or 1 mm (**C**). (**B**,**D**) Primary root length (**B**) and primary root diameter (**D**) of the seedlings shown in (**A**,**C**). Each column is the average of 30 seedlings and the bars indicate ± SD. The asterisks indicate significant differences compared to 0 mM NaCl (* *p* < 0.05, ** *p* < 0.01, *** *p* < 0.001, Student’s *t*-test).

As the belowground organ of the plant, root structure is shaped by soil bio-physico-chemical properties [29,30]. Salinity is a major abiotic stress that represses plant growth and crop yields, and the roots of young rice seedlings are highly salt-sensitive organs [31]. To dissect the effect of salt stress on root growth, we treated rice seedlings with different concentration of NaCl. Salt stress significantly inhibited primary root elongation and promoted root swelling, and the effect increased with increasing salt concentrations (Figure 1A–D), suggesting that salt stress has a dual role in rice primary root growth, i.e., the restriction of primary root elongation and the swelling of the root tip.

**Figure 2:**
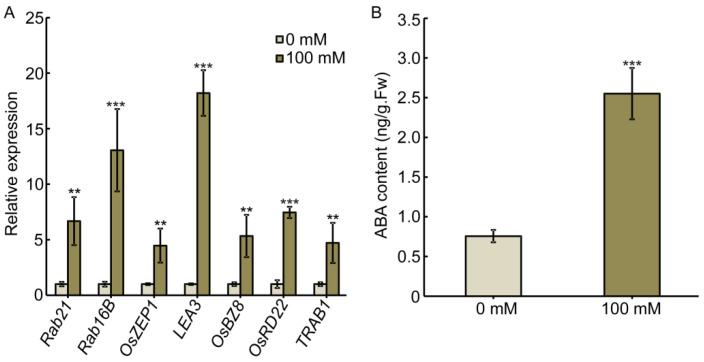
Salt stress promotes ABA accumulation in primary roots. (**A**) Expression of ABA-responsive genes in 4-day-old seedling root. RNA was isolated from roots for quantitative polymerase chain reaction (qPCR) analysis. Three biological replicates were performed with similar results, and a representative experiment is shown. Samples were collected from three independent experiments. Bars indicate ± SD. (**B**) ABA content in 4-day-old primary root with or without 100 mM NaCl treatment. Bars indicate ± SD of three biological replicates. The asterisks indicate significant differences compared to 0 mM NaCl (** *p* < 0.01, *** *p* < 0.001, Student’s *t*-test).

The phytohormone ABA regulates plant development in response to adverse environments, including salt stress [32,33]. We therefore asked whether salt-regulated root growth in rice requires the function of ABA. To address this question, we detected the expression of ABA-responsive genes [34] and ABA content in primary roots with or without salt treatment. Our results showed that the expression of ABA-responsive genes is significantly induced by salt stress (Figure 2A). Correspondingly, ABA content significantly accumulated in primary roots after salt treatment (Figure 2B). These results indicate that salt stress promotes the accumulation of ABA in primary roots, resulting in short and swollen primary roots.

**Figure 3:**
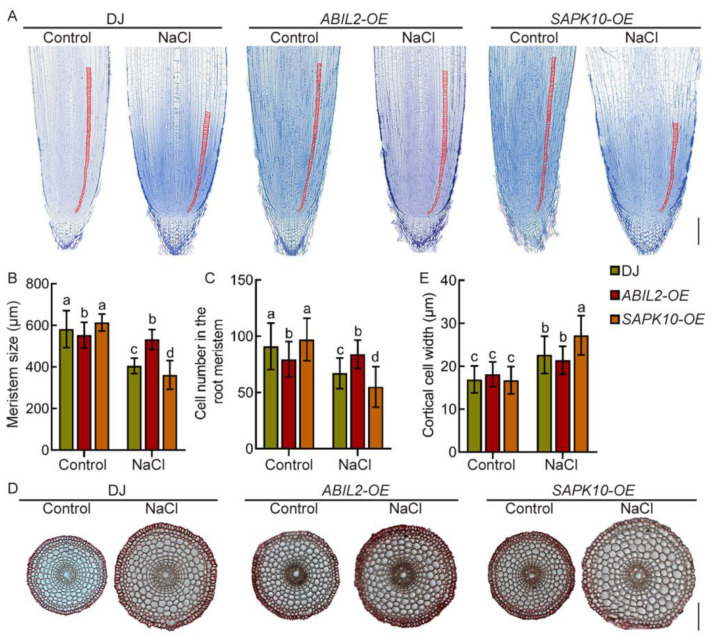
Salt inhibits cell proliferation and promotes cortical cell expansion in the root meristem, dependent on ABA signaling. (**A**,**D**) Longitudinal sections (**A**) and cross-sections (**D**) of primary root tips (lower) of 4-day-old *DJ*, *ABIL2-OE*, and *SAPK10-OE* seedlings treated with 0 mM and 100 mM NaCl. Red lines delimit the meristem size (i.e., the distance between the quiescent center and the transition zone). Bar = 100 μm. (**B**,**C**,**E**) Length of root meristematic zone (**B**), cortical cell number in the root meristem zone (**C**), and cortical cell diameter (**E**) of the corresponding seedlings indicated in (**A**,**D**). In (**B**,**C**,**E**), data are means ± SD (*n* ≥ 10 independent seedlings). Different letters indicate significant differences (*p* < 0.05, one-way ANOVA with Tukey’s test).

To provide further proof that salt-regulated root growth in rice requires the function of ABA, we used rice lines overexpressing *SAPK10* (a positive regulator of rice ABA signaling) or *OsABIL2* (a negative regulator of rice ABA signaling) for further analyses. Salt treatment significantly inhibited primary root elongation (~60%) and promoted primary root swelling (~19%) in wild type Dongjing (DJ) roots, with a milder phenotype in *ABIL2-OE* seedling roots and a more pronounced phenotype in *SAPK10-OE* seedling roots (Figure S1A–D). Microscopic analysis of root tip longitudinal and cross sections further demonstrated that salt treatment reduced the meristem size (~30%) and the cortical cell number (~22%), and increased the cortical cell diameter (~24%) in DJ roots. This tendency was weakened in *ABIL2-OE* seedling roots, but enhanced in *SAPK10-OE* seedling roots (Figure 3A–D). These results indicate that salt inhibits cell proliferation and promotes the radial expansion of cortical cells in the root meristem, thus resulting in short and swollen primary roots, and this effect largely depends on the ABA signaling pathway.

### 2.2. ABA Inhibits Cell Proliferation and Promotes Radial Expansion of Cortical Cells in the Root Meristem

ABA has biphasic effects on primary root growth, depending on its concentrations, environmental conditions, developmental contexts, genotypes, and plant species [12,18,19]. To investigate the effect of ABA on primary root growth in rice early seedlings, we treated rice varieties with different genetic backgrounds, including three *Japonica* cultivars (Nipponbare (Nip), Zhonghua 11 (ZH11), and Hwayoung (HY)) and three *Indica* cultivars (Zhenshan#97 (ZS97), Yangdao#6 (YD6), and IR29), with various concentrations of ABA. Exogenous application of 0.1 μM ABA significantly inhibited the elongation of primary roots (~23–31%) in all tested genotypes and the inhibitory effect increased with the increase of ABA concentrations (Figure S2A,B), suggesting that the inhibitory effects of ABA on primary root elongation is common in rice early seedlings and that ABA inhibits primary root elongation in a dose-dependent manner.

**Figure 4:**
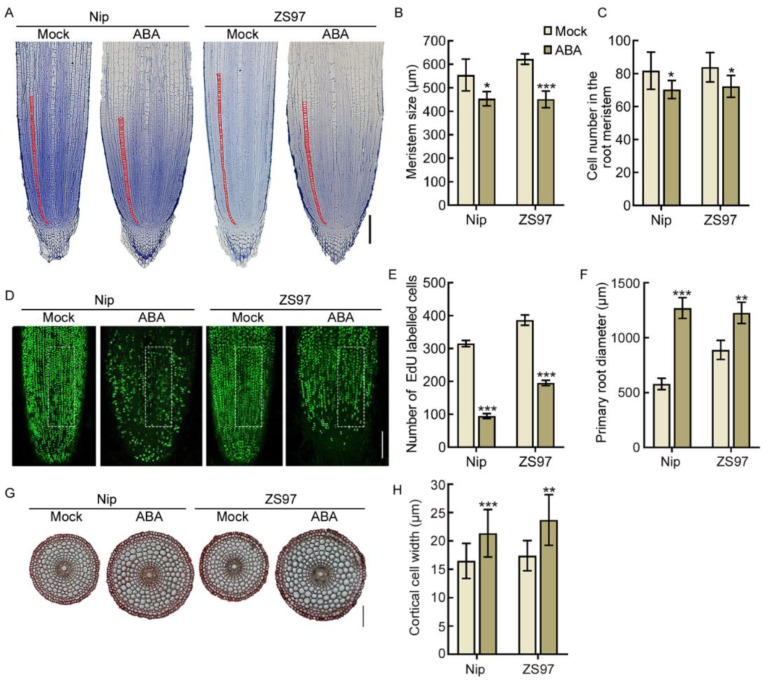
ABA inhibits cell proliferation and promotes cortical cell expansion in the root meristem. (**A**) Longitudinal sections of primary root tips (lower) of 4-day-old Nip and ZS97 seedlings treated with 0 μM (Mock) and 1 μM ABA. Red lines delimit the meristem size (i.e., the distance between the quiescent center and the transition zone). Bar = 100 μm. (**B**,**C**) Length of root meristematic zone (**B**) and cortical cell number in the root meristem zone (**C**) of the corresponding seedlings indicated in (**A**). (**D**) EdU-labelled cells in the root meristem of 4-day-old Nip and ZS97 seedlings treated with 0 μM (Mock) and 1 μM ABA. Bar = 100 μm. (**E**) Number of EdU-labelled cells in (**D**). Quantification was performed in the unit area (white box) of the root tip. (**F**) Primary root diameter of the corresponding seedlings indicated in (**A**). (**G**) Cross-sections of primary root tips (lower) of 4-day-old Nip and ZS97 seedlings with or without 1 μM ABA treatment. Bar = 100 μm. (**H**) Diameter of cortical cells in (**G**). In (**B**,**C**,**E**,**F**,**H**), data are means ± SD (*n* ≥ 10 independent seedlings). The asterisks indicate significant differences compared to mock (* *p* < 0.05, ** *p* < 0.01, *** *p* < 0.001, Student’s *t*-test).

Root growth is regulated through the activity of root meristems. The root growth rate correlates with the cell number in the root meristem, as this determines the number of cells that can differentiate in a given time [10,35]. To determine whether ABA inhibits primary root elongation through modulating cell proliferation in the root meristem, we analyzed the median longitudinal sections of root apices of 4-day-old wild-type rice. ABA treatment significantly reduced the root meristem size (~11–33%) and the cortical cell number (~21–37%) in the root meristem (Figure 4A–C), suggesting that ABA inhibits cell proliferation in the root meristem. This finding was further confirmed by 5-ethynyl-2′-deoxyuridine (EdU) staining (an indicator of cell proliferation) [10], as ABA significantly reduces the number of EdU labeled cells in the root meristem (~49–69%) (Figure 4D,E). These results indicate that ABA inhibits cell proliferation in the root meristem, thus restricting primary root elongation.

Hormones control organ growth by regulating specific growth processes in distinct tissues [9,36], and accumulating investigations in *Arabidopsis* show that ABA mainly functions on cortical cells [37]. To evaluate the effect of ABA on cortical cells in the root tips of rice, we measured the diameter of the primary roots of 4-day-old rice seedlings with or without ABA treatment. The roots underwent a doubling in width after ABA treatment (Figure 4A,F), indicating that ABA promotes root tip swelling. To further dissect the mechanism of ABA on root swelling, cross-sections of the root tip were produced (Figure 4G). The cortical cell width was significantly increased after ABA treatment (~33–40%) (Figure 4H). These results indicate that ABA triggers cortical cell radial expansion, thus leading to the swelling of the root tip.

### 2.3. ABA Induces the Expression of EXPANSIN Genes

**Figure 5:**
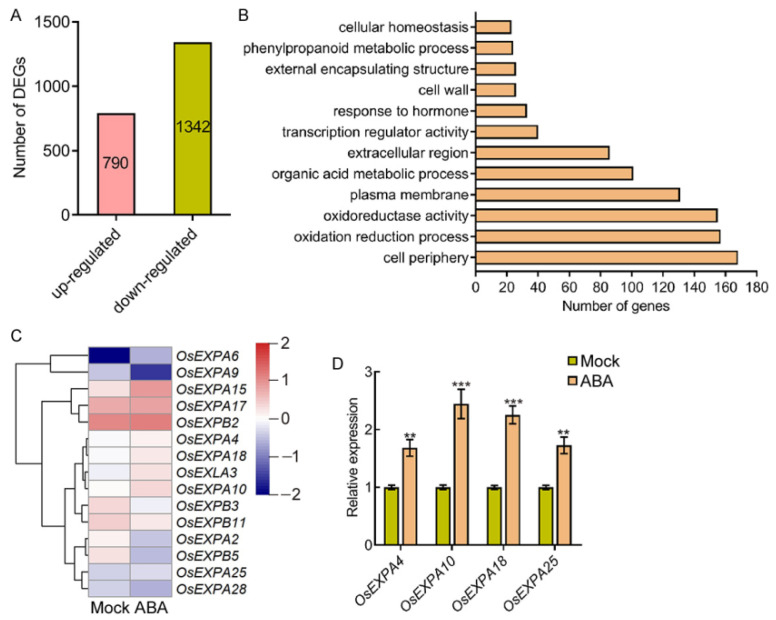
ABA induces the expression of *EXPANSIN* genes. (**A**) Number of DEGs up- and down-regulated in the root treated with ABA. (**B**) GO term analysis of ABA up- and down-regulated genes. (**C**) Heat-map of the microarray expression profiles of *EXPANSIN* genes. (**D**) Quantitative polymerase chain reaction (qPCR) analysis of *EXPANSIN* genes in 4-day-old seedling roots treated with or without 1 μM ABA for 3 h. Three biological replicates were performed with similar results, and a representative experiment is shown. Samples were collected from three independent experiments. Bars indicate ± SD. The asterisks indicate significant differences compared to Mock (** *p* < 0.01, *** *p* < 0.001, Student’s *t*-test).

To elucidate the molecular network underlying ABA-regulated root elongation and swelling, we treated 4-day-old seedlings with or without ABA and performed transcript profiling of the roots of these seedlings. RNA-seq analyses showed that in total 2132 differentially expressed genes (DEGs) were identified after ABA treatment, including 790 up-regulated DEGs and 1342 down-regulated DEGs (Figure 5A, Table S1). Gene ontology (GO) enrichment analysis showed that these ABA up- and down-regulated DEGs were involved in the cell periphery, the oxidation reduction process, and the organic acid metabolic process (Figure 5B). Notably, two classes of genes, involved in the response to hormone and cell wall organization or biogenesis, were enriched after ABA treatment (Figure 5B), suggesting that ABA may control root growth by regulating genes involved in cell wall organization or biogenesis.

EXPANSINs are thought to be a key wall-loosening factor that have long been implicated in the control of plant growth processes as modulators of cell wall extensibility [38]. Therefore, we selected *EXPANSIN* (*EXP*) and *EXPANSIN-LIKE* (*EXL*) for further analysis. The heat-maps show that the expression of *EXP* and *EXL* genes is differentially regulated after ABA treatment (Figure 5C). Quantitative polymerase chain reaction (qPCR) analysis showed that ABA induces the expression of *OsEXPA4*, *OsEXPA10*, *OsEXPA18*, and *OsEXPA25*, which are preferentially expressed in young seedling roots [39] (Figure 5D). These results indicate that ABA stimulates the expression of *EXPANSIN* genes to control root elongation and swelling.

### 2.4. Regulation of ABA in Primary Root Growth Depends on Its Signaling Pathway

**Figure 6:**
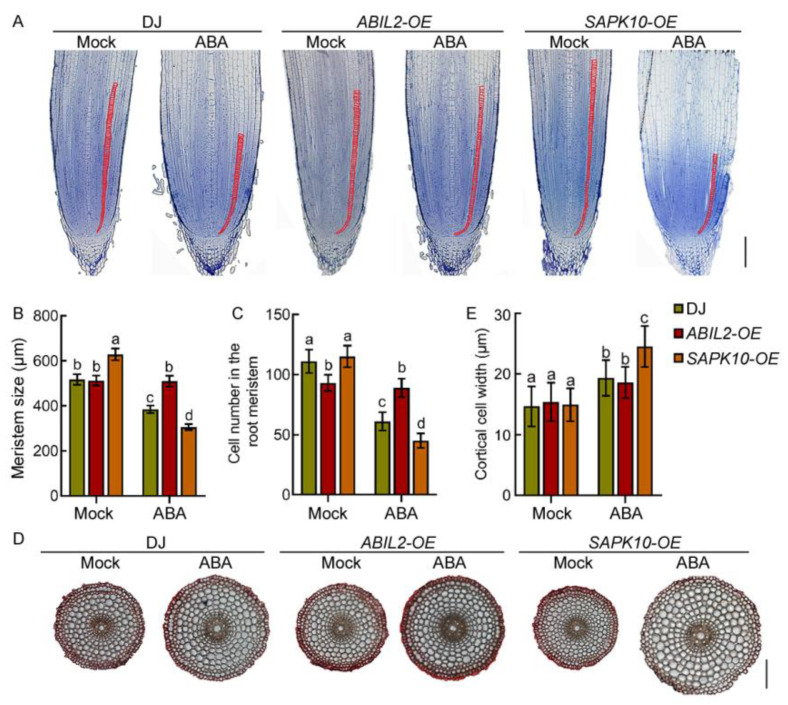
The regulation of ABA in cell proliferation and expansion in the root meristem depends on ABA signaling. (**A**,**D**) Longitudinal sections and cross-sections of primary root tips (lower) of 4-day-old Dongjing (DJ), *ABIL2-OE*, and *SAPK10-OE* seedlings with or without ABA treatment. Red lines delimit the meristem size (**A**). Bar = 100 μm. (**B**,**C**,**E**) Length of root meristematic zone (**B**), cortical cell number in the root meristem zone (**C**), and diameter of cortical cells (**E**) of the corresponding seedlings indicated in (**A**,**D**). Data are means ± SD (*n* ≥ 10 independent seedlings). Different letters indicate significant differences (*p* < 0.05, one-way ANOVA with Tukey’s test).

ABA exerts its effects largely through signaling [12,40]. To investigate whether ABA regulates primary root elongation and swelling through the major ABA signaling components, we treated *SAPK10-OE* and *OsABIL2-OE* lines with ABA. Our results showed that ABA treatment significantly inhibited primary root elongation (~41%) and promoted primary root swelling (~61%) in DJ roots (Figure S3A–D), with a milder phenotype in *ABIL2-OE* seedling roots and a more pronounced phenotype in *SAPK10-OE* seedling roots (Figure S3A–D). Microscopic analysis of root tip longitudinal sections further demonstrated that ABA treatment reduced the meristem size (~26%) and cortical cell number (~45%) and increased cortical cell diameter (~30%) in DJ roots. This tendency was weakened in *ABIL2-OE* seedling roots, while it was enhanced in *SAPK10-OE* seedling roots (Figure 6A–E), demonstrating that the modulation of ABA in cell proliferation and cell expansion in the root meristem are primarily mediated by the ABA signaling pathway.

**Figure 7:**
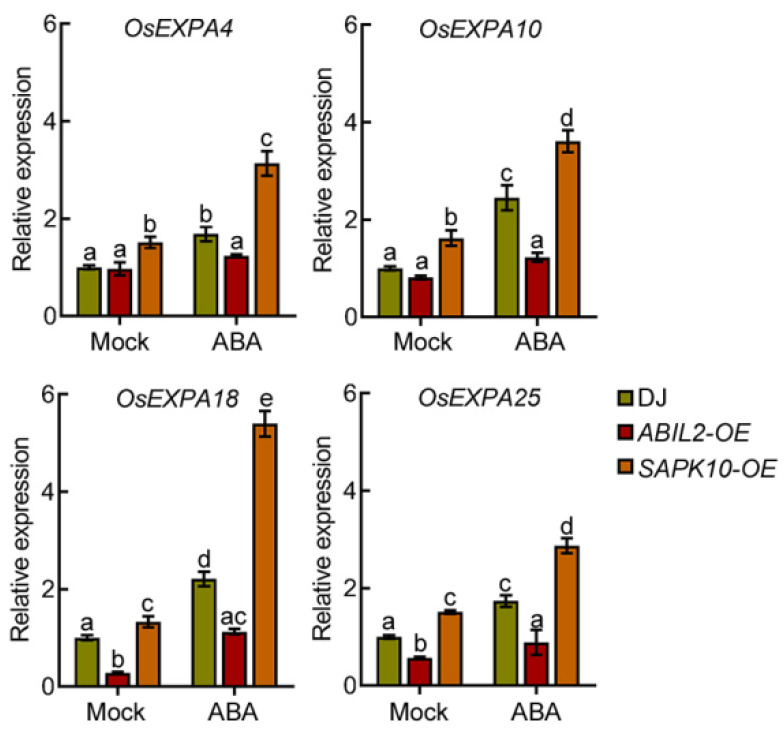
The induction of ABA in the expression of *EXPANSIN* genes depends on ABA signaling. Quantitative polymerase chain reaction (qPCR) analysis of *OsEXPA4*, *OsEXPA10*, *OsEXPA18*, and *OsEXPA25* in 4-day-old Dongjing (DJ), *ABIL2-OE*, and *SAPK10-OE* seedling roots treated with or without 1 μM ABA for 3 h. Three biological replicates were performed with similar results and a representative experiment is shown. Samples were collected from three independent experiments. Bars indicate ± SD. Different letters indicate significant differences (*p* < 0.05, one-way ANOVA with Tukey’s test).

Next, we detected the expression of *EXPANSIN* genes in 4-day-old DJ, *ABIL2-OE*, and *SAPK10-OE* seedling roots with or without ABA treatment. Our qPCR analyses showed that the expression of *EXPANSIN* genes was decreased in *ABIL2-OE* seedling roots but increased in *SAPK10-OE* seedling roots in the absence of ABA, and ABA-induced *EXPANSIN* gene expression was weakened in *ABIL2-OE* seedling roots but enhanced in *SAPK10-OE* seedling roots (Figure 7). These results suggest that ABA induces *EXPANSIN* gene expression dependent on the ABA signaling pathway.

## 3. Discussion

The primary root is the basic component of the root system and plays a key role in early seedling growth in rice [41]. Its growth is precisely regulated by environmental stimuli and phytohormones [29,42,43]. Elucidating the underlying mechanism of primary root growth is a prerequisite for improving the root system of rice and cultivating high-yield and stress-resistant rice. In *Arabidopsis*, ABA has biphasic effects on primary root growth. Typically, low concentrations of ABA stimulate primary root growth, whereas high concentrations inhibit it [19]. In the present study, we demonstrated that salt stress has a dual role in rice primary root growth, namely, inhibiting root elongation and promoting root apical swelling. Further investigations showed that salt stress promotes the accumulation of ABA in the primary root, thereby activating *EXPANSIN* genes to inhibit cell proliferation and promote cell expansion in the root meristem, ultimately inhibiting primary root elongation and promoting primary root swelling. Thus, our findings deepen our understanding of the role of ABA in controlling primary root growth in response to salt stress.

Root growth is determined by the coordination of cell proliferation, differentiation, and expansion [9,10,44]. In *Arabidopsis*, low concentrations of ABA promote primary root development through maintaining the activity of the quiescent center (QC) and suppressing the differentiation of stem cells and their daughter cells in the root meristem [45]. Here, we showed that 0.1 μM ABA can significantly inhibit primary root elongation in rice, whereas this concentration of ABA promotes root elongation in *Arabidopsis* [19], indicating that the effect of ABA on primary root growth is distinct in different plant species. Microscopic analysis indicates that ABA inhibits cell proliferation and promotes cell expansion in the root meristem, resulting in a short and swollen primary root. Deep rooting is favorable for the acquisition of water and nitrogen from the subsoil [46], whereas swollen root is advantageous for water and nutrient flux and root branching [47]. Thus, the dual role of ABA on root growth is conducive for achieving optimal growth and higher grain production under adverse conditions in rice.

EXPANSINs are unique plant cell wall proteins that are involved in cell wall modifications underlying many development processes and stress responses in plants [24,48,49,50]. Under most conditions, the EXPANSINs participating in these biological processes involve plant hormones [28,51,52]. In the present study, we revealed that ABA promotes radial expansion of cortical cells in the root meristem, resulting in a swollen primary root. Transcriptome analysis showed that genes involved in cell wall organization or biogenesis were regulated by ABA, including *EXPANSIN* genes. Further investigations showed that *OsEXPA4*, *OsEXPA10*, *OsEXPA18*, and *OsEXPA25* were induced by ABA, and this induction depends on the ABA signaling pathway. This finding deepens our understanding of the role of ABA in root growth. Further investigations will be required to determine how *OsEXPAs* are regulated by ABA to promote root swelling.

Salt stress is a major environmental stress that restricts the growth and yield of crops [3]. Rice is considered the most salt-sensitive cereal crop and the roots of young rice seedlings are highly salt-sensitive organs that limit plant growth [31]. Dissecting the molecular mechanism of rice roots in response to salt stress is an important objective for rice breeding. Previous studies have demonstrated that salt stress induces ethylene biosynthesis in the primary root and that ethylene directs ABA to inhibit primary root elongation [43,53,54], indicating that ABA might be involved in salt-regulated root growth. In the present study, we found that salt treatment inhibits primary root elongation and promotes primary root apical swelling, in a dose-dependent manner, mimicking the effect of ABA treatment of the primary root. Correspondingly, the expression of ABA-responsive genes and ABA content in the primary root also increased after salt treatment. Taken together, based on our present data and previous reports, we propose a modulatory model that salt stress stimulates ethylene biosynthesis in the primary root, leading to the activation of the expression of *MHZ4/5*, consequently promoting ABA accumulation in the primary root and thereby inhibiting cell proliferation and promoting cell expansion in the root meristem, ultimately resulting in a short and swollen primary root. Our results deepen our understanding of ABA-induced root growth with changing environmental stimuli, providing knowledge for breeders that will facilitate the selection of new rice cultivars suitable for saline land.

## 4. Materials and Methods

### 4.1. Plant Materials and Growth Conditions

Rice varieties Nipponbare (Nip), Zhonghua 11 (ZH11), Hwayoung (HY), IR29, Yangdao#6 (YD6), and Zhenshan#97 (ZS97) were preserved in the laboratory. The rice overexpression lines *OsABIL2-OE* or *OsSAPK10-OE* were described previously [11]. For propagation, crossing, and investigation of agronomic traits, the plants were cultivated at the Experimental Station of the Chinese Academy of Agricultural Sciences in Beijing, China, during the natural growing seasons.

### 4.2. ABA and Salt Treatments and Analysis of Root Growth

Germinated rice seeds were placed on a stainless steel sieve in an air-tight 10 L plastic box. Seeds were treated with 6 L of water containing various concentrations of ABA or NaCl. The seedlings were grown under a 14 h light/10 h dark cycle at 28 °C for 4 days. At the end of the period, the roots were scanned and root length was measured from digitized images using Image J software.

### 4.3. Quantitative Real-Time PCR (qPCR)

Total RNA was extracted from (approximately) 0.5 g of 4-day-old root tissue with an Ultrapure RNA Kit (CWBIO, Beijing, China, CW0581S) according to the manufacturer’s instructions. ~2 μg total RNA was reverse transcribed to cDNA with HiScript II Q RT SuperMix (Vazyme, Nanjing, China, R223-01) according to the manufacturer’s instructions. The qPCR was performed on a Bio-Rad iQ5 system as previously described [55]. The rice *OsActin1* gene was used as an internal standard to normalize gene expression. The qPCR primers are listed in Table S2.

### 4.4. Histological Observations

Rice tissue sections were generated as previously described [43]. Root apices (~5 mm) of 4-day seedlings were fixed overnight at 4 °C in 2.5% glutaraldehyde in 0.2 M sodium phosphate buffer, pH 7.2, and washed three times for 30 min in the same buffer. The root samples were fixed for 4 h in 1% OsO_4_ in 0.1 M sodium phosphate buffer, pH 7.2, and washed for 30 min in the same buffer. The samples were dehydrated in a gradient ethanol series and embedded in Spurr’s resin. Semithin sections (1 μm thick) were produced using a Leica EM UC7 microtome and stained in 0.1% methylene blue for 3 to 5 min at 70 °C. Finally, the samples were rinsed with distilled water and visualized under a microscope (Nikon ECLIPSE Ni, Tokyo, Japan).

### 4.5. 5-Ethynyl-2′-Deoxyuridine (EdU) Staining

EdU staining was performed using an EdU kit (Guangzhou, China, C10310, Apollo 488) according to the manufacturer’s protocol. Briefly, roots of 4-day-old rice seedlings were immersed in 50 μM EdU solution for 12 h, and then fixed for 30 min in 4% paraformaldehyde, followed by 30 min of incubation with Apollo. The samples were next hand-sectioned longitudinally and EdU images of the sections were then captured with a ZEISS LSM980 confocal microscope (Jena, Germany) using an excitation wavelength of 488 nm. Quantification of numbers of EdU-stained cells was performed in a selected area with a length of 300 μm and a width of 100 μm in the root meristem.

### 4.6. Transcriptome Analysis

4-day-old seedlings were treated with or without 1 μM ABA for 3 h, and the mRNA was extracted and purified from 2 μg total RNA by using a Dynabeads mRNA purification kit (Invitrogen, Waltham, MA, USA). The mRNA was then subjected to RNA-seq library construction for the transcriptome experiments using the ultra-RNA library prep kit (NEB, Ipswich, MA, USA). Multiplex paired-end adapters were used for multiplex libraries. The RNA-seq libraries were quantified using a bioanalyzer (Agilent, Santa Clara, CA, USA), then sequenced (paired-end, 100 bp each) by the Illumina genome analyzer (Hiseq 2000; Illumina, San Diego, CA, USA). After removing the adaptor and low-quality reads, clean reads were mapped to the rice genome MSU7.0 reference using TopHat and analyzed using Cufflinks according to Trapnell et al. [56]. A Poisson dispersion model of the fragment was used to conduct statistical analysis (FDR < 0.05) and responsive genes were identified by fragments per kilobase per million reads (FPKM), requiring more than a twofold change between two samples. Three biological replicates were used and their repeatability and correlation were evaluated by the Pearson’s Correlation Coefficient [57].

### 4.7. Measurement of ABA

ABA content was detected using 4-day-old seedling roots treated with or without 100 mM NaCl. Roots (200 mg) were ground into powder in liquid nitrogen. ABA was determined by an LC-ESI-MS/MS system (HPLC, Shim-pack UFLC SHIMADZU CBM30A system; MS, Applied Biosystems 6500 Triple Quadrupole) at Metware. Each series of experiments was performed in biological triplicates.

## Data Availability

Sequence data from this article can be found in the Rice Genome Annotation Project website, avaiable online: http://rice.plantbiology.msu.edu/, under the following accession numbers: *OsActin1*, Os03g50885; *OsEXPA4*, Os05g39990; *OsEXPA10*, Os04g49410; *OsEXPA18*, Os03g06040; *OsEXPA25*, Os03g06010; *TRAB1*, Os08g36790; *OsBZ8*, Os01g46970; *LEA3*, Os05g46480; *OsZEP1*, Os04g37619; *Rab21*, Os11g26790; *Rab16B*, Os01g24050; *OsRD22*, Os01g53240. accessed on 1 August 2021).

## Supplementary Materials

The following are available online at https://www.mdpi.com/article/10.3390/ijms221910892/s1.
