# Supplementary material for: Salt Stress Promotes Abscisic Acid Accumulation to Affect Cell Proliferation and Expansion of Primary Roots in Rice
Source: Int J Mol Sci. 2021 Oct 8;22(19):10892. doi: 10.3390/ijms221910892 (PMC8509385; doi:10.3390/ijms221910892)
Supplement: Supplementary file 1 [file ijms-22-10892-s001.zip › Supplementary Files/Supplemental Figures.pdf]

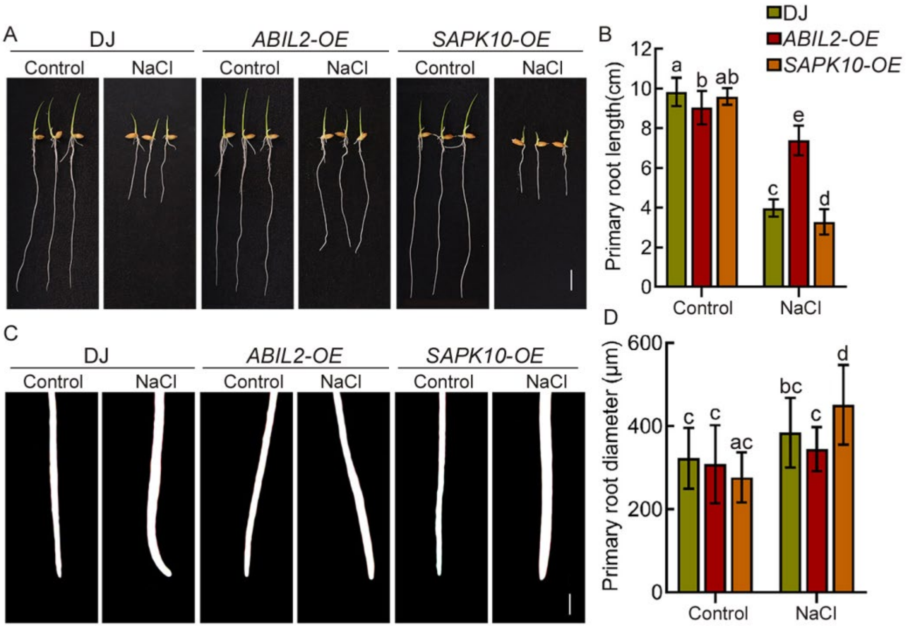

Figure S1. Salt inhibits root elongation and promotes root swelling is dependent on ABA signaling. (**A** and **C**) Root phenotypes of Dongjing (DJ), *ABIL2-OE*, and *SAPK10-OE* seedlings treated with or without 100 mM NaCl aqueous solution. Bar = 1cm. (**B** and **D**) Primary root length (**B**) and primary root diameter (**D**) of the seedlings shown in (**A**) and (**C**). Each column is the average of 30 seedlings, and bars indicate ± SD. The experiment was repeated at least three times with similar results. Different letters indicate significant differences ($P < 0.05$, one-way ANOVA with Tukey's test).

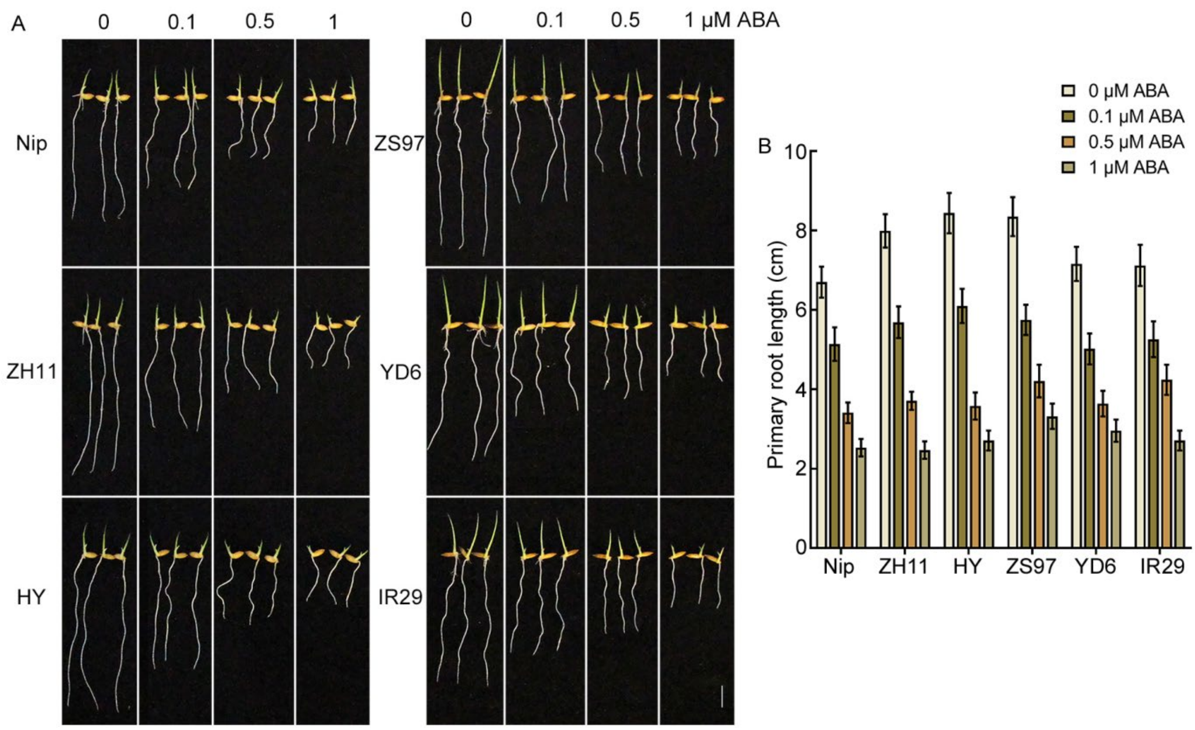

Figure S2. ABA inhibits primary root elongation in rice early seedlings. (**A**) Root phenotypes of various *japonica* (Nip-Nipponbare, ZH11-Zhonghua#11, HY-Hwayoung) and *indica* (ZS97-Zhenshan#97, YD6-Yangdao#6, IR29) cultivars treated with 0, 0.1, 0.5 and 1 μM ABA solution. Bar = 1cm. (**B**) Primary root length of the seedlings shown in (**A**). Each column is the average of 30 seedlings, and bars indicate ± SD. The experiment was repeated at least three times with similar results.

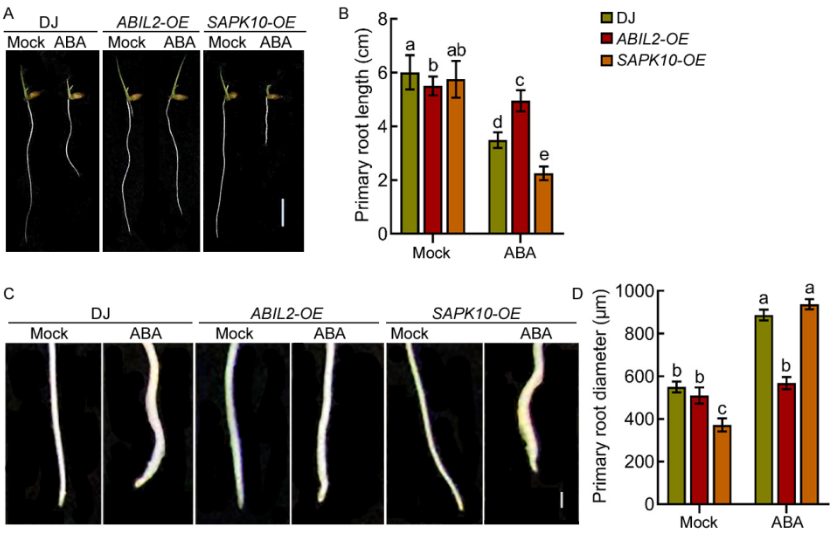

Figure S3. ABA-regulated primary root elongation and swelling depends on ABA signaling pathway.

(A and C) Root phenotypes of Dongjing (DJ), *ABIL2-OE* and *SAPK10-OE* seedlings with or without ABA treatment. Rice seedlings were grown under normal conditions for 3 days in the absence (mock) or presence of 1 μM ABA. Bar = 1 cm (A) or 1 mm (C). (B and D) Primary root length (B) and primary root diameter (D) shown in (A) and (C). Each column is the average of 30 seedlings, and bars indicate ± SD. Three biological replicates were performed, with similar results. Different letters indicate significant differences ($P < 0.05$, one-way ANOVA with Tukey's test).
