# Supplementary material for: Salt Stress Promotes Abscisic Acid Accumulation to Affect Cell Proliferation and Expansion of Primary Roots in Rice
Source: Int J Mol Sci. 2021 Oct 8;22(19):10892. doi: 10.3390/ijms221910892 (PMC8509385; doi:10.3390/ijms221910892)
Supplement: Supplementary file 1 [file ijms-22-10892-s001.zip › Supplementary Files/Supplemental Table S2.pdf]

Table S1. Primers used in this study

Primer name	Primer sequence (5' - 3')
OsActin1-F	GACCTTGCTGGGCGTGAT
OsActin1-R	GTCATAGTCCAGGGCGATGT
OsEXPA4-F	AGCCAGCGTTTAGACACCAT
OsEXPA4-R	GTGCCATACCCTTGGCTGTA
OsEXPA10-F	ATGACCTCAAGTACTGCCTC
OsEXPA10-R	CTTCTCGAAGGACTGGATGG
OsEXPA18-F	CATGGGGAACATTGTCCTGC
OsEXPA18-R	CGTACAGATTCCCGTACCCG
OsEXPA25-F	ATCTACAACGTCGTGCCCAG
OsEXPA25-R	AGCTTGAGATCATGCAGACGA
TRAB1-F	GTCAATGATGCTGCTGCTG
TRAB1-R	CCTTGCCCGCTGAATCC
OsRD22-F	TTGGTTGCTTATGCTCTA
OsRD22-R	CACCCGTATCATCTACTA
OsBZ8-F	CACCTAATGATAGACCTAGCGAACAAG
OsBZ8-R	TCATCCTTAGTTGGCACTTCAGTTAC
LEA3-F	GTGATGTGTACTGATGATGTT
LEA3-R	CAAATGCGGGCTTTAGG
OsZEP1-F	CGGTTGGTGATGATGCTATAC
OsZEP1-R	CTGAATAAGTTGCTTCTGATTGC
Rab16B-F	AGCTCCAGCTCGTCGTCTGA
Rab16B-R	GCCAGTGTTCCCCATCATCT
Rab21-F	CGAGCGCAATAAAGGAAAAA
Rab21-R	AGACACGGTCCGTACTGGAGAA
